# Association between systemic osteoporosis and mandibular condyle characteristics: A comparative imaging study

**DOI:** 10.6026/973206300220304

**Published:** 2026-01-31

**Authors:** Parnavi Mishra, Annette M Bhambal, Harish Rao, Sudhanshu Saxena, Preeti Nair, Shivakumar G.C

**Affiliations:** 1 Department of Oral Medicine and Radiology, People's College of Dental Sciences and Research Centre, Bhopal, Madhya Pradesh, India; 2 Department of Orthopaedics, People's College of Medical Sciences and Research Centre, Bhopal, Madhya Pradesh, India; 3 Department of Dentistry, MGM Medical College and Hospital, Jamshedpur, Jharkhand, India

**Keywords:** Osteoporosis, mandibular condyl, bone density, cone-beam computed tomography, morphology

## Abstract

Osteoporosis is a generalized skeletal disease, which is associated with lowered bone density and changed bone structure. Therefore, it is of interest to compare and evaluate the bone mineral density (BMD) and morphology of the mandibular condyle of patients with osteoporosis and no osteoporosis using the imaging method. The sample size of 30 patients (over 40 years old) was selected at random and split into two equal groups i.e. Control (non-osteoporotic) and Experimental (Osteoporotic). Systemic bone density was measured with ultra-heel bone densitometer and the condylar bone density parameters and morphological changes were measured with the Cone-beam computed tomography (CBCT). Osteoporotic subjects showed low bone density of the condyles than that of non-osteoporotic controls.

## Background:

The progressive alterations of physiology that are linked to aging are numerous and most of them are minor but of clinical significance. The decrease in skeletal strength is one of these factors, which is a major cause of morbidity in the elderly. The bone mass is highest at the age of early adulthood and then falls. It is this loss of bone mass and bone strength that forms the basis of osteoporosis, a generalized skeletal disorder that results in the body becoming brittle and prone to bone fractures [1]. The pathogenesis of osteoporosis is based on a complex of hormonal, cellular and metabolic processes. According to the World Health Organization, osteoporosis can be defined as a skeletal systemic disease that is characterized by loss of bone mass and microarchitecture, which leads to high risk of fractures [2]. Although osteoporosis is a disease that occurs all over the body, the craniofacial appearances of the disease, especially in the mandible, became a subject of concern. Despite the fact that the majority of the researches were dedicated to the mandibular body, the condylus is comparatively under-researched. The mandibular condyle which is mainly made up of trabecular bone with a thin cortical layer also displays continuous remodelling that is affected by functional and metabolic stimuli. It is sensitive to systemic metabolic changes, which indicates its possible usefulness as an early warning of osteoporotic changes [3]. The evaluation of skeletal microarchitecture has been significantly improved in recent years due to the development of the imaging technologies. The cone Beam Computed Tomography (CBCT) allows three-dimensional and high-resolution publications of the temporomandibular joint and condylar morphology and provides valuable information on the localized bone alteration [4]. Simultaneously, heel ultrasonography is a non- radiative, non-invasive measurement of peripheral bone mineral density. These imaging modalities when used in combination provide a thorough model of the analysis of cranio facial and systemic skeletal well-being [5]. Therefore, it is of interest to compare mandibular condyle bone mineral density (via CBCT grayscale values) and morphology between osteoporotic and non-osteoporotic TMD patients using heel ultrasonography for systemic validation, establishing condyle as a novel dental imaging marker for early osteoporosis screening.

## Materials and Methods:

## Patient selection:

A cross-sectional, single blind comparative study to determine the qualitative and quantitative features of the mandibular condyle of the osteoporotic and non-osteoporotic individuals was carried out in the Department of Oral Medicine and Radiology Peoples College of Dental Sciences and Research Centre, Bhopal. The inclusion criteria were that the participants needed to be over 40 years of age, have the clinical symptoms of temporomandibular disorders (TMD) and to be diagnosed based on the Diagnostic Criteria of Temporomandibular Disorders (DC/TMD). The study enrolled only the subjects who were in written informed consent. Other metabolic bone diseases were excluded because the participants had the history or clinical evidence of such diseases. Included were those who were already undergoing treatment of osteoporosis or had already used anti-resorptive therapy (bisphosphonate or calcitonin) or anabolic therapy (fluoride or parathyroid hormone). Those who had cancer history irrespective of the occurrence of bone metastasis, and women who were using hormone replacement therapy (HRT) were also not included in the study. Persons, who met the inclusion criteria, were further split into two groups (Group I 30 osteoporotic patients with TMD / Study group and Group II 30 non- osteoporotic patients with TMD / Control group).

## Assessment of osteoporosis:

The systemic and regional bone changes were measured on all the 60 participants.

## Systemic bone assessment:

Systemic BMD was qualitatively assessed using Heel Ultra Densitometry (Furuno CM-300). The diagnosis of osteoporosis was made based on the ICD-10-CM Code M81.0, 2024. T-score of -1.0 and above were considered non-osteoporotic, which means that the bone was normal. Those who had T-scores between 1 -2.5 were treated as Osteopenic which is the loss of bone mass and a medium risk of developing osteoporosis. General bone density was reduced and high risk of fractures was observed in individuals with T-scores that are equal to or less than -2.5 which were subsequently categorized as being osteoporotic [6].

## Regional bone assessment:

CBCT (CS 9600) was used to measure the characteristics of condylar bone in two parameters namely, qualitative and quantitative.

[1] Qualitative evaluation of morphology or Morphological Bone Changes (MBC): Condylar shape was determined using sagittal sections using a slice thickness of 1.1 mm. The scanner was configured to a maximum output of 120 kV and 5 mAs with an exposure time of 40 seconds, the voxel size was 150-3 and field of view was 818 cm 8 cm. The 4 variations (Ovoid, Angled, Bird Beaked and Flat) (7) on the right and left condylar head were rated individually by 2 examiners. The status of the subjects on osteoporosis was hidden when they were examined.

[2] Structural Bone Changes or quantitative assessment: In the quantitative measures, the bone density of the mandibular condyle was measured in coronal section with a slice of 1.1mm and 8 x 8cm field of view. Circle notation tool was used to circle the inner cortical surfaces of the condyle and the bone density at the centre of the circle was measured with the help of density measurement tool of CBCT software, in the right and left condyle. (8). The region of interest (ROI) was measured in Greyscale Value (GSV) to get mean density values. The data received was inputted in Microsoft Excel 365 windows and results were studied statistically.

## Statistical analysis:

Various frequencies, percentages, mean, standard deviation (SD), minimum and maxima of variables were computed. The Shapiro-Wilk test of normality revealed that the data of age had a normal distribution, and condylar density (GSV) did not have a normal distribution. Accordingly, an unpaired t-test was applied to compare the mean ages and Mann-Whitney U to compare the condylar density between osteoporotic and non-osteoporotic groups. To make comparisons between the categorical variables, Chi-square test of Pearson was utilized; whereby any of the cells with an expected frequency below 5 was compared using Chi-square test with Yates correction. The p-value that was found to be less than 0.05 was taken to be significant. All the analyses were made in SPSS software, version 23.0 (IBM Corp., Armonk, NY, USA).

## Results:

The mean ± SD age of the osteoporotic group was 55.00 with a SD of 9.79 years and the non-osteoporotic group was slightly less with a mean ± SD age of 51.43 with SD of 7.76 years. The age range of both groups was the same and was 39.00 to 74.00. Group I consisted of 22 females against 8 males with an unpaired t-test showing non significance difference in mean age between the two groups (t =1.564, p > 0.05), indicating that there was no significant difference with regard to the distribution of age between the two groups. Systemic BMD evaluation was qualitatively performed using Heel Ultra Densitometry (Furuno CM-300). Osteoporosis diagnosis was made with reference to the ICD-10-CM Code M81.0 of 2024. T-Score of the osteoporotic (group) fell between 2.5 to 3.15 which is in line with the WHO criteria of osteoporosis.

**Figure 1:**
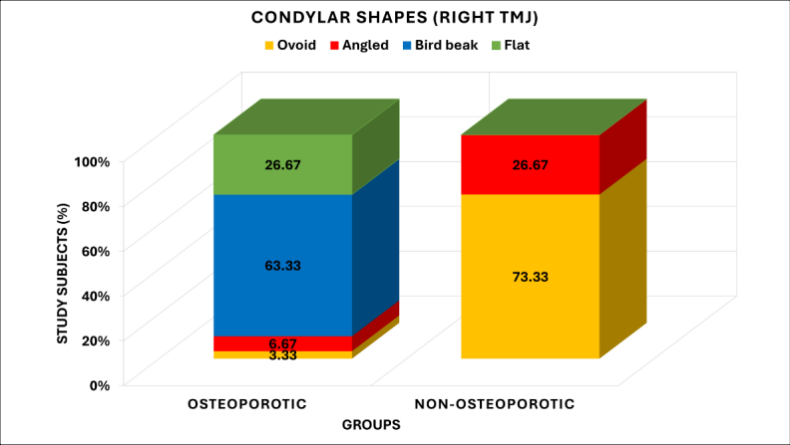
Comparison of condylar shapes of right TMJ between osteoporotic and non- osteoporotic groups

**Figure 2:**
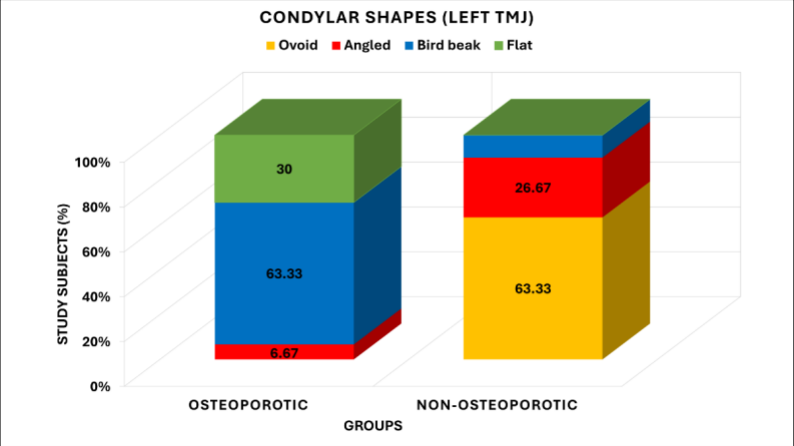
Comparison of condylar shapes of left TMJ between osteoporotic and non- osteoporotic groups

Figure 1 illustrates the size of condyles of right temporomandibular joint between osteoporotic and non-osteoporotic groups. One participant (3.33) had an ovoid shape, 2 (6.67) an angled shape, 19 (63.33) a bird beak shape and 8 (26.67) a flat condylar shape in the osteoporotic group. Conversely, 22 people (73.33%) of the non-osteoporotic group had ovoid condyles, 8 (26.67%) angled and none of the birds had either bird beak or flat forms. The analysis of chi-square test showed that the difference in the distribution of condylar shapes between the groups is very highly significant (Yates χ^2^ = 43.069, df =3, P <0.001) and there was a clearly different pattern in the distribution of condylar shapes between the groups. Figure 2 illustrate the difference in condylar forms of left temporomandibular joint (TMJ) between the osteoporotic and non-osteoporotic. The osteoporotic group indicated that 0 individuals (0.00) had an ovoid shape, 2 (6.67) an angled shape, 19 (63.33) a bird beak shape and 9 (30.00) a flat condylus. On the other hand, the non-osteoporotic group consisted of 19 participants (63.33%) who had ovoid condylar shape, 8 (26.67%) participants with an angled shape, 3 (10.00) participants with a bird beak shape and none with a flat shape. The analysis of chi-square test showed that the distribution of condylar shapes was very highly significant between the groups (Yates -2 = 36.891, df =3, P <0.001), and the pattern of left condylar shapes distribution between the groups was not the same.

**Figure 3:**
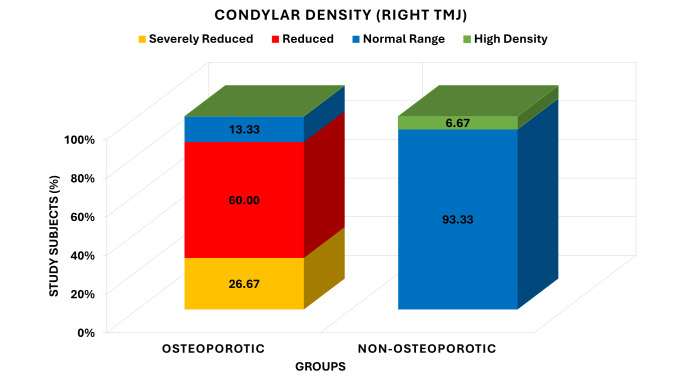
Comparison of grades of condylar density on right TMJ between osteoporotic and non- osteoporotic groups

Figure 3 shows the difference in the condylar density grade on the right temporomandibular joint in the osteoporotic and non-osteoporotic groups. Amongst the osteoporotic, 8 (26.67) participants were severely reduced, 18 (60.00) reduced and 4 (13.33) within the normal range of condylar density; no one was high density. On the other hand, the non-osteoporotic segment had no participants that were severely reduced or reduced in the condylar density, 28 (93.33) had a normal range of density, and 2 (6.67) had high density. The analysis of the chi-square test showed the significantly different pattern of the condylar density between the groups in the case of Yates 002 = 39.212, df =3, P <0.001 which demonstrates that the distribution of the condylar density is significantly different between the groups.

**Figure 4:**
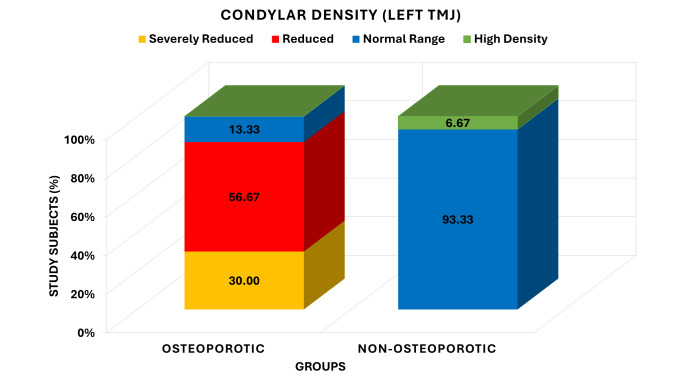
Comparison of grades of condylar density on left TMJ between osteoporotic and non- osteoporotic groups

The data in Figure 4 represent the descriptive results of the comparison of condylar density grades on the left temporomandibular joint between non-osteoporotic and osteoporotic groups. In the osteoporotic group, 9 (30.00) participants had severely diminished condylar density, 17 (56.67) had diminished density, and 4 (13.33) had density that was within the normal limit, and none was high density. Conversely, the non-osteoporotic group had no severely reduced or reduced density of the condylar in the group, 28 (93.33) had the normal range density and 2 (6.67) had the high density. The analysis by Chi-square test showed a very highly significant difference between the distribution of the condylar density grades of the groups (Yates 2 =39.201, df=3, P <0.001), a markedly different pattern of condylar density between the groups was observed.

## Discussion:

Osteoporosis is a global illness in which the bone mass is lost as well as the bone architecture which leads to bone fragility and risks of fractures. It is projected that osteoporosis is likely to rise tremendously in the future due to population aging. Mostly osteoporosis is found in postmenopausal women and older men. The National Institutes of Health Consensus Development Panel on Osteoporosis defines osteoporosis as a skeletal disorder, which is described as loss of bone strength causing increased risk of fracture. Additionally, osteoporosis by the definition of the criteria of the World Health Organization (WHO) is a bone mineral density (BMD), which is at least 2.5 standard deviation (SD) below the average level of healthy young women (T-score ≤ 2.5 SD) [8]. The symptoms of osteoporosis are not limited to axial or appendicular locations but also spread to the craniofacial ones. The maxilla and mandible are especially prone to the effects of osteoporosis due to their high metabolic rate and functional requirements. Changing bone mineral density in these areas can undermine or jeopardize oral health, influence the stability of prosthetics, and cause morphological alterations in the craniofacial complex [9]. Several studies have been done to assess the mandibular body and alveolar process in relation to their potential in assessing osteoporosis, however, the mandibular condyle is not that explored in the same context. One such area of interest is mandibular condylar region, since it has biological and structural similarity with the trabecular rich peripheral bones, such as the calcaneus or the heel bone. The condylus and the calcaneus have a thick trabecular structure and are highly bones remodelling active making them the most sensitive indicators of systemic bone metabolism. It is on these similarities that alterations in the bone mineral density of the mandibular condyle can be a viable reflection of osteoporotic changes at peripheral locations including the heel and can also give useful information about the overall bone health and early osteoporosis [10]. Osteoporotic condyles used bird-beak shapes (63) and flat shapes (2630) in our study, and non-osteoporotic condyles were ovoid (6373). Such results indicate that osteoporotic bone fragility in the system to remodel and degenerate condylar morphology. These morphological alterations could be the adaptive modifications to the weakening of the trabecular structures and cortical thinning linked with systemic osteoporosis [7, 11]. These findings support and build on the finding that systemic osteoporosis is associated with characteristic condylar morphological changes in imaging and confirms that metabolic bone changes are reflected as transitions in shape that are significant in populations with osteoporosis, toward ovoid forms and flat-bird beaks [12]. Bird beak morphology was more prevalent in both sides (63.33), suggesting that bilateral systemic bone fragility triggers remodelling. Nevertheless, there was total lack of ovoid shapes (0%) in the left condylus and an increased ratio of flat condylus (30% vs. 26.67) in the left as opposed to the right. This implies that further degenerative remodelling occurs on the left condylar side, and more ovoid morphology is replaced by flat morphology. The chi square values endorse very significant differences on both sides but the shift in the pattern is more pronounced on the left condyle. These results are consistent with others who report that lateralization of functions as evidenced by habitual chewing dominance could result in more mechanical loads on the preferred side of the body, typically the left, thus hastening bone resorption and morphological changes during the occurrence of systemic osteoporosis. Such reports uphold the notion that regular unilateral mastication may cause increased mechanical stress which in turn can cause accelerated bone resorption and morphological adjustment, particularly in people with weakened bone quality in the case of osteoporosis [13, 14-15]. Other studies have further proposed that minor differences in disc movement patterns, can also result in side specific susceptibility of the condylar bone [16]. Lack of teeth in people with osteoporosis has a great effect on the biomechanical situation of the temporomandibular joint. Edentulism undermines occlusivity and diminishes the vertical dimension hence changes mandibular loading dynamics. It causes a redistribution of the forces on the condylar surfaces, which facilitates bone remodelling. Research has proven that edentulous condyles tend to experience morphological changes such as flattening, surface anomalies, and reduced trabecular density more than dentate individuals. The morphological changes are exacerbated by the combined effect of systemic skeletal frailty and disturbed functional loading of edentulous osteoporotic patients which frequently results in a sharp change in ovoid to flat or bird-beak condylar shapes. Consequently, edentulous condition is an important modifier in the evolution of condylar erosion related with osteoporosis [17, 18].

There was an increase in the susceptibility of the lateral pole of the mandibular condyle of osteoporotic subjects to early resorptive alterations as a result of a combination of biomechanical, anatomical, and radiographic factors. Mechanically, the effect of lateral mandibular excursions is asymmetric loading of the temporomandibular joint (TMJ), the working side condyle rotating with little translation and the balancing side condyle translating in the forward-downward-medial fashion (Bennett movement). These active compressive forces on the lateral pole of the working condyle specifically on the lateral condylar path and they add to local strain and remodelling. The Fisher angle [normal about 5 to 7 degrees] also shows the separation of the protrusive and lateral paths, which further confirms the hypothesis about the lateral pole bearing more rotational and compressive forces during the time of mastication [19]. The anatomical studies of cone-beam computed tomography (CBCT) have found that the lateral pole was always found to be less cortically thick than the medial, thereby making it more susceptible to osteoporotic loss and weakening of the trabecular [20]. Radiographically, imaging examination of TMJ inner derangement and fractures of the condylar appearance shows that resorptive alterations usually commence in the posterior section of the lateral pole usually advancing toward typical flattening and bird-beak deformity. All these results point towards the lateral pole to being a site of degenerative remodelling in patients with osteoporosis, akin to both systemic bone weakness and focal biomechanical stress [21]. The resorption pattern that was found in the mandibular condyle parallels the results that were obtained in other skeletal areas susceptible to osteoporosis. Zhao *et al.* study showed that bone-resorption lesions of the osteonecrosis of the femoral head are located mainly in the lateral and central pillars, which were exposed to high mechanical forces and are structurally determined by the collapse. This is similar to the susceptibility of the lateral pole of mandibular condylar bone which also has concentrated functional loads and reduced the cortical thickness. This type of similarities shows the systemic character of osteoporotic bone loss and implies that craniofacial imaging can provide additional information regarding the overall skeletal health [22]. The decrease in bone mineral density in osteoporotic people causes the thinning of trabecular structure and cortical frailty, which stimulate the remodellation of highly trabecular areas including condylar head. The anterior-superior surface of the condyle instead of maintaining its physiological ovoid contour proceeds to experience progressive resorptive alterations, with residual areas being relatively preserved. This asymmetry causes the formation of pointed anterior projection which is radiographically identified as bird-beak morphology. Putri *et al.* also support these results by carrying out an observational descriptive study that compared the density and morphology of the condylar head in osteoporotic and non-osteoporotic subjects. Their findings showed that there were significant changes in the structure of the condyles in osteoporotic individuals and further confirmed the relationship between the fragility of the bones in the body and the degeneration of the craniofacial aspect locally [7]. The quantitative data of the mean grayscale values (GSV) were significantly reduced and significantly different between osteoporotic and non-osteoporotic patients suggesting the susceptibility of mandibular condyle to the general bone loss. These results are consistent with Kanneppady *et al.* and Poiana *et al.* as they also found similar reduction in condylar trabecular density in osteoporotic and postmenopausal individuals also using CBCT. Their findings indicated the usefulness of jaw bone density measurement in the detection of osteoporosis and the grayscale values derived by use of CBCT have a correlation between the mandibular bone and the systemic bone mineral density (BMD) which mandibular bone is a possible site of early detection of osteoporosis [23, 24]. The results of Gungor *et al.* also contribute to the perception that the value of grayscale or density of the mandible bone, including the condyle, is an indicator site that can be used to identify osteoporosis early on [25]. Both osteoporotic condyles had a great presence in density between the range of 6089 GSV (60) and significant rate within the below range of 60 GSV (2730). The left condyle is slightly more severe 30% in the <60 GSV range than on the right (26.67) indicating more advanced trabecular loss. By comparison, non-osteoporotic condyles fell in the 90139 GSV ranges (93.33%), with a low percentage of 140 densities (6.67). The chi square findings affirm that there is a very highly significant difference between the osteoporotic and non-osteoporotic groups on both sides, which supports the fact that the condylar density is a sensitive indicator of bone fragility in the body.

The evidence of reduction in condylar density of the patient with osteoporosis is an indication of the systemic pathophysiology of osteoporosis where a greater activity of osteoclastic cells and a defective bone formation are observed, resulting in an imbalance that leads to progressive thinning of the trabecular, the elevation in cortical porosity and the general skeletal fragility [26]. These changes are especially sensitive to the mandibular condyle which is made of mainly trabecular bone that is surrounded by a thin cortical shell. There is structural deterioration of the condylar head, which is manifested by a decrease in radiodensity, morphological changes, and loss of biomechanical integrity. The trabecular network in the condyle turns out to be disorganized and sparse, and the cortical margins become thinner putting the body at a higher risk of resorptive changes and deformities [3]. It is also observed that bone loss at the mandibular condyle normally starts at the center of the trabecular area as opposed to the marginal areas of the cortical bone [27]. This central zone which is composed of a majority of metabolically active trabecular bone is more sensitive to the systemic changes like osteoporosis, and more sensitive to an earlier and acute resorption process caused by high levels of osteoclastic activity and lower bone formation. Conversely, the peripheral cortical shell is denser and slower to remodel and is, therefore, relative spared in the early stages of bone loss. With the degradation of the internal trabecular network, structural integrity of the condylar feature is impaired and morphological changes such as flattening or bird-beak projections are common especially when the condyles are loaded functionally. Cone beam CT and panoramic radiography imaging studies always demonstrate a low radiodensity of the center of osteoporotic condyles, and it is sharply contrasted with the comparatively untouched periphery, which supports the trend of central-first resorption [28]. Anatomical structure of the mandibular condyle and its exposure to constant functional loading during mastication makes this part a sensitive area to detect bone loss in the system. Such changes are not only localized but are a sign of skeletal degeneration. This research does show some limitations that can impact on the interpretation of the findings and further implementation of the findings. To begin with, the sample size is relatively small and limits the extrapolation of the findings to work with a wide variety of populations. Also, no uniformed CBCT grayscale reference values make it difficult to compare across studies the bone density, which may affect consistency and reproducibility. Moreover, the CS 9600 CBCT system applied in this paper is incapable of generating true Hounsfield Units (HU), which limits its ability to measure absolute bone mineral density (BMD) and decreases its diagnostic accuracy in the diagnosis of osteoporosis. The results of the research indicate that the mandibular condylar bone is often not included in the list of bones assessed in the health of the body, although the mandibular condylar bone can also be used to assess skeletal frailty by ensuring changes in both bone density and morphology occur simultaneously. In order to improve the diagnostic reliability, future studies need to include bigger cohorts and longitudinal study designs that would provide a set of standardized CBCT grayscale thresholds of osteoporosis screening. In addition, the existing imaging platform (CS 9600 CBCT) does not have the power to transform grayscale values to real Hounsfield Units (HU) and thus is not useful in quantifying bone mineral density in a more accurate way.

## Conclusion:

Distorted condylar morphology and bone density may have a great impact on the biomechanics of the temporomandibular joint (TMJ), exposing them to a range of functional disorders. The atrophy of the trabecular integrity and cortical thinning causes the condylar structures to fail at supporting the masticatory forces, which results in asymmetrical distribution of stress and poor articulation in the glenoid fossa. Such structural alterations can cause displacement of the discs, pain in the joints, low masticatory performance, which eventually presents as internal derangement and chronic TMJ dysfunction.
